# Necrotizing pneumonia: an emerging problem in children?

**DOI:** 10.1186/s41479-017-0035-0

**Published:** 2017-07-25

**Authors:** I. Brent Masters, Alan F. Isles, Keith Grimwood

**Affiliations:** 1 Department of Respiratory and Sleep Medicine, Lady Cilento Children’s Hospital, South Brisbane, QLD Australia; 2 School of Medicine and Menzies Health Institute Queensland, Gold Coast campus, Griffith University, Building G40, Southport Gold Coast, QLD Australia; 3 Departments of Infectious Diseases and Paediatrics, Gold Coast Health, Southport Gold Coast, QLD Australia

**Keywords:** Necrotizing pneumonia, Child, *Streptococcus Pneumoniae*, *Staphylococcus aureus*, Panton-Valentine leukocidin, Empyema, Bronchopleural fistulae

## Abstract

**Background:**

In children, necrotizing pneumonia (NP) is an uncommon, severe complication of pneumonia. It is characterized by destruction of the underlying lung parenchyma resulting in multiple small, thin-walled cavities and is often accompanied by empyema and bronchopleural fistulae.

**Review:**

NP in children was first reported in children in 1994, and since then there has been a gradual increase in cases, which is partially explained by greater physician awareness and use of contrast computed tomography (CT) scans, and by temporal changes in circulating respiratory pathogens and antibiotic prescribing. The most common pathogens detected in children with NP are pneumococci and *Staphylococcus aureus*. The underlying disease mechanisms are poorly understood, but likely relate to multiple host susceptibility and bacterial virulence factors, with viral–bacterial interactions also possibly having a role. Most cases are in previously healthy young children who, despite adequate antibiotic therapy for bacterial pneumonia, remain febrile and unwell. Many also have evidence of pleural effusion, empyema, or pyopneumothorax, which has undergone drainage or surgical intervention without clinical improvement. The diagnosis is generally made by chest imaging, with CT scans being the most sensitive, showing loss of normal pulmonary architecture, decreased parenchymal enhancement and multiple thin-walled cavities. Blood culture and culture and molecular testing of pleural fluid provide a microbiologic diagnosis in as many as 50% of cases. Prolonged antibiotics, draining pleural fluid and gas that causes mass effects, and maintaining ventilation, circulation, nutrition, fluid, and electrolyte balance are critical components of therapy. Despite its serious nature, death is uncommon, with good clinical, radiographic and functional recovery achieved in the 5–6 months following diagnosis. Increased knowledge of NP’s pathogenesis will assist more rapid diagnosis and improve treatment and, ultimately, prevention.

**Conclusion:**

It is important to consider that our understanding of NP is limited to individual case reports or small case series, and treatment data from randomized-controlled trials are lacking. Furthermore, case series are retrospective and usually confined to single centers. Consequently, these studies may not be representative of patients in other locations, especially when allowing for temporal changes in pathogen behaviour and differences in immunization schedules and antibiotic prescribing practices.

## Background

Necrotizing pneumonia (NP) is an uncommon, but severe complication of pneumonia. It is characterized by progressive pneumonic illness in a previously healthy child despite appropriate antibiotic therapy, and runs a protracted clinical course [1]. The diagnosis is made by chest imaging studies showing one or more small thin-walled cavities within areas of pulmonary consolidation. Pathological examinations from autopsies or resected lung specimens reveal pulmonary inflammation, alveolar consolidation and thrombosis of intrapulmonary vessels with accompanying necrosis and multiple small cavities [1, 2]. It is speculated that reduced blood flow from thrombosed vessels decreases antibiotic concentrations within the affected lung tissue, leading to persistent infection and further destruction of pulmonary tissue. NP lies on a spectrum between pulmonary abscess and pulmonary gangrene, and is accompanied frequently by empyema and bronchopleural fistulae (BPF) [2].

First reported in adults with severe pneumonia in the 1940s, NP was not described in children until 1994 when a small case series of four patients was published [3]. Since then, descriptions of NP have included individual case reports [4–12] and small retrospective series of 2–18 cases [13–27] with few publications involving larger numbers of affected children. Although still regarded as uncommon, the incidence of NP (as with other more common forms of complicated pneumonia, parapneumonic effusion [PPE] and empyema) may be increasing [18, 22, 28–32]. NP may be complicating 0.8–7% of all cases of community-acquired pneumonia (CAP) [29, 30, 33–35] and up to 20% of those with empyema admitted to tertiary pediatric hospitals [18]. Therefore, it is timely to review NP in children. To do so, the authors searched PubMed for articles published in English up to and including 31 January 2017. The search terms were ‘necrotizing pneumonia’ and ‘children’ in combination, in titles and abstracts, and the search was focused upon larger case series and publications from the last decade. The reference lists of articles identified by this search strategy were also searched and relevant articles retrieved. The search found eight retrospective reviews of ‘all cause’ NP involving 20–80 childhood cases [28, 30, 31, 33, 35–38]. In each of these, the diagnosis of NP was confirmed in one of three ways: radiographically, requiring solitary or multiple thin-walled cavities within areas of consolidation, while specifically excluding thick-walled, fluid or air-filled cavities with well-defined enhancing rims identifying pulmonary abscesses [28, 30, 31, 33, 36, 37]; by direct observation of lung necrosis during thoracoscopy [38]; or by inference from the report [35]. Similarly, another six retrospective reports describing NP in 10–57 children with proven *Streptococcus pneumoniae* (pneumococcal) necrotizing pneumonia (PNP) were identified where there was limited evidence of overlapping cases from the same institution and sufficient information on the radiographic criteria to differentiate these cases from those with empyema alone [25, 29, 32, 34, 39, 40]. However, two of these case series [29, 34] included abscess within their definition of NP. These 14 studies are summarized in Tables 1 and 2 and form the basis of our review.

**Table 1:** Retrospective studies in children with necrotizing pneumonia and where participant numbers exceeded 20 subjects

Study	Subjects	Causes	Imaging results	WBC count and inflammatory markers	Management	Course	Comments
Wong et al. [36] Taiwan. Single tertiary center. Almost 5 yr. review July 1995–March 1999.	No. = 21 12 (57%) males. Mean (SD) age = 2.4 (1.3) yrs. No co-morbidities.	Pathogen detected in 9 (43%) cases. Blood culture +ve in 2 (9.5%) cases - *S. pneumoniae* 2 Hib 1 Pleural culture +ve in 5 (24%) cases - *S. pneumoniae* 2 *S. aureus* 2 Hib 1 Four-fold increase in *M. pneumoniae* serum antibody titres and negative bacterial cultures *n* = 2 (9.5%).	All had CT scans confirming NP diagnosis. PPE 20 (95%) - 6 (30%) loculated empyema BPF 8 (38%) Abscess 4 (19%).	NR.	Antibiotics and chest tube drainage only 9 (43%). Thoracoscopy (VATS) 10 (48%) - pulmonary segmental resection 2. Antibiotics alone 2 (9.5%).	Medically treated: - mean (SD) fever duration before hospitalization 6.8 (3.3) days - mean (SD) total fever duration 9.5 (3.5) days - mean (SD) hospital LOS 21.7 (5.5) days Plus surgery: - mean (SD) fever duration before hospitalization 14.9 (9.6) days - mean (SD) total fever duration 16.5 (8.5) days - mean (SD) hospital LOS 30.0 (8.0) days *P < 0.05.	Fever (95%), cough (52%) and respiratory distress (43%) were the most common presenting symptoms. The 10 children undergoing VATS had more severe disease than those medically treated and had failed conservative treatment. At 6 mths, 19/21 (90%) had normal chest radiographs, 2 had small residual pneumatoceles.
Hacimustafaoglu et al. [37] Turkey. Single tertiary center. 4 yr. review of prospective collected data.	No. = 36. Mean (SD) age = 3.8 (3.3) yrs. (Range 9 mths-14 yrs). No co-morbidities.	Pleural fluid *S. pyogenes* 1 *S. pneumoniae* 3. All had received antibiotics before admission to hospital.	CT scans required for diagnosing NP. Lungs involved - right 20 (55%) left 12 (33%) both 4 (11%). PPE 34 (94%) - septated 80% loculated 33% BPF 20 (55%).	Mean (SD) peripheral WBC count = 19.3 (8.7) × 10^9^/L. Mean (SD) CRP = 136 (117) mg/L. Pleural fluid (mean (SD)) -glucose 1.3 (1.2) mmol/L. LDH 793 (682) IU/L.	Thoracotomy 24 (67%)	Mean (SD) symptom duration before presentation = 11.9 (8.5) days. Mean (SD) febrile days = 8.9 (4.3) after hospitalization. Mean (SD) hospital LOS = 26 (9) days. Mortality rate 5.5%.	Diagnosis of NP by CT scan made on average 6 days after admission and 17 days after symptom onset.
Sawicki et al. [28] United States. Single tertiary center. 15 yr. review January 1990–February 2005.	No. = 80. 45 (53%) males. Median (IQR) age = 3.6 (2.4–6.2) yrs. Most were healthy - 14 asthma 1 CNLD 1 cerebral palsy 2 PID. Cases increased from 3 per yr. in 1990–93 to 14 per yr. in 2003–4.	Pathogen detected, in 38 cases (48%), 19 from testing pleural fluid in 50 cases. *S. pneumoniae* 18 (13 by culture) MSSA 5 MRSA 3 *Fusobacterium* 1 *P. aeruginosa* 1 CONS 4 *S. anginosus* 6 group. 54 (68%) had received antibiotics before admission to hospital.	CT scans required for diagnosing NP. -PPE 69 (86%) -BPF 10 (13%).	Mean (SD) peripheral WBC count = 18.4 (8.9) × 10^9^/L. Mean (SD) CRP = 133 (93) mg/L. Mean (SD) haemoglobin = 10.4 (1.6) g/L. Mean (SD) serum albumen = 20 (16) g/L. Pleural fluid (mean (SD) or median (IQR)) - pH 7.08 (0.33) -glucose 0.5 (0.1–3.6) mmol/L -LDH 2810 (1413–9530) IU/L -Cell count 9.6 (1.2–56) × 10^6^/L -Pleural neutrophils 70 (23)%.	Procedures for PPE – 69 (86%). Chest tube drainage only 47 (68%). Chest drainage + surgery 16 (23%). Thoracentesis only 6 (9%).	Intensive care 25 (31%); 1 ECMO. Median (IQR) pleural drainage days = 6 (4.5–9.5). Median (IQR) febrile days = 6 (3–9). Median (IQR) hospital LOS = 12 (9–17) days. Median (IQR) antibiotic days = 28 (20–40). No deaths.	Fever (96%) and cough (84%) were main symptoms, at a mean onset of 9 days before presentation. 8 (10%) readmitted within 2wks of being discharged; 64 seen 6mths post discharge, all well, 12 had PFTS, 3 had mild obstructive and 1 mild restrictive defects. CXR and CT scans markedly improved and near normal at 6 mths.
Macedo et al. [38] Brazil. Single tertiary center. 6 yr. review July 2002–June 2008.	No. = 24. 12 (50%) males. Median age 2 yrs. (Range 1–9 yrs).	Pleural fluid culture *-S. pneumoniae* 6 -MSSA 3 *-P. aeruginosa* 1.	CXR, CT and US performed. PPE 24 (100%) PTX 11 (46%) BPF 16 (67%).	NR.	All underwent thoracoscopy. Prior chest drainage had been undertaken in 19 (79%) of the children.	Median chest drainage time = 18 days (Range 1–30 days). Median hospital LOS = 19 days. 7–49 days). No deaths reported.	Subjects were children undergoing thoracoscopy for a loculated empyema and/or PTX and in whom a diagnosis of NP was made when a cavity containing necrotic debris was found in the lung tissue. None underwent a thoracotomy.
Jester et al. [35] UK. Single tertiary center. 10 yr. review January 2000–May 2010.	No. = 20. 13 (65%) males. Median age 2.5 yrs. (Range 0.8–6.8 yrs). All were referred because of BPF complicating empyema.	Pleural fluid culture or PCR *S. pneumoniae* 10 (serotype 3, *n* = 3) *S. aureus* 1 All had received antibiotics for a median duration of 7 days (Range 2–13) prior to referral	All had pre-operative CT scans. Lobes involved - right - lower 10 (50%) upper 4 (20%) - left - lower 3 (15%) upper 3 (15%). BPF was found on presentation in 11 (55%), in 4 (20%) during thoracoscopy, and in 5 (25%) post-operatively.	NR.	All had a serratus anterior muscle digitation flap inserted.	Median post-operative fever was 2 days (Range 1–3 days). Median duration of chest tube drainage post-operatively was 7 days (Range 5–15 days). Median hospital LOS = 9 days (Range 7–28 days). No deaths reported.	All were referred because of persistent signs of empyema with clinical deterioration and diagnosis of NP complicated by BPF. The median follow-up was 4 yrs. (Range 2 mths-10 yrs). All had normal thoracic volumes on CXR without signs of scoliosis.
Lemaitre et al. [30] France. Single tertiary center. 5 yr. review May 2006–April 2011.	No. = 41. 22 (54%) males. Median age 1.2 yrs. (Range 1 mth- 16 yrs). 1 child had sickle cell anemia. The rate of NP complicating CAP doubled from 4.5% in 2006–2009 to 9% in 2009–2011.	Blood/pleural/BAL fluid cultures +ve in 21 (51%) cases. - MSSA 12 - MRSA 1 (All *S. aureus* strains PVL + ve) *-S. pneumoniae* 7 (serotypes 3, 7F, 19A) *-F. nucleatum* 1 (14 yr. old with sickle cell anemia). 18 (44%) had received antibiotics before hospital admission.	CXR and CT scans performed. CXR revealed necrosis on admission in 11 (27%) cases. CT scan performed on 23 (56%) children and detected NP in 12 not apparent on CXR. PPE 26 (63%) PTX 8 (20%)	Median peripheral WBC count = 20,000 × 10^6^/L (Range 1700–44,300). Median CRP = 141 mg/L (Range 5–434).	Procedures for PPE – 26 (63%). - Thoracentesis 10- VATS 6	Median symptoms before presentation = 4 days (Range 1–10 days). Nursed in intensive care 7 (17%). Median febrile days in hospital = 7 days (Range 1–25 days). Median hospital LOS = 16 days (Range 7–43 days). Median antibiotic days = 42 days (Range 31–60 days). No deaths.	NP diagnosis made by a combination of clinical signs of lung infection and radiographic signs of NP with multiple thin-walled cavities within a region of homogenous alveolar consolidation. The *S. pneumoniae* serotypes identified in this series are not included in PCV7 available in France at the time of the study.
Krenke et al. [33] Poland. Single tertiary center. 5 yr. review April 2008–July 2013.	No. = 32. 14 (44%) males. Median age 4 yrs. (Range 1–10 yrs). Most were healthy - 2 asthma 1 obesity and hypertension 1 atrial septal defect.	Blood cultures +ve in 6 (19%) cases. Pleural fluid cultures +ve in 7 (23%) children. *- S. pneumoniae* 8 - MSSA 2 -*S. milleri* 1- CONS 1 -*S. maltophilia* 1. All had received antibiotics before admission to hospital.	CXR ± CT scans performed. Lungs involved - right 18 (56%) left 10 (31%) both 4 (13%). PPE 31 (97%) PTX 2 (6%) BPF 8 (25%).	Median (IQR) peripheral WBC count = 21.3 (15.2–24.1) × 10^9^/L. Median (IQR) CRP = 182 (152–241) mg/L. Median (IQR) haemoglobin = 8.9 (8.4–9.9) g/L. Median (IQR) serum albumen = 25 (23.5–27.5) g/L. Pleural fluid (median (IQR)) - pH 7.3 (7.0–7.5) glucose 2.8 (1.8–3.1) mmol/L -LDH 8670 (2828–14,254) IU/L.	Procedures for PPE – 31 (97%). Thoracentesis only 2 (6%). Chest tube drainage 28 (90%), including urokinase in 25 (81%). VATS 1 (3%).	Median febrile days before presentation = 6 days (Range 1–10 days). Median febrile days in hospital = 9 days (Range 0–22 days). Median (IQR) chest drainage time = 8.6 (6–11.25) days. Median hospital LOS = 26 days (Range 13–44 days). Median (IQR) antibiotic days = 28 (22.5–32.5) days. No deaths.	Rate of NP amongst all CAP admissions was 32/882 (3.6%). Fever (94%), tachypnea (90%) and cough (77%) most common symptoms. 5 mths later all children had normal physical exams and CXRs showed complete or almost complete resolution of pleural and lung abnormalities.
Erlichman et al. [31] Israel. Three tertiary centers. 10 yr. review 2001–2010.	No. = 29. 19 (66%) males. Median age 3.1 yrs. Case rates increased from <0.5/100,000 in 2001–2003 to ~2/100,000 in 2009–2010.﻿	Blood cultures +ve in 3/29 (10%) cases - *S. pneumoniae* 3. Pleural fluid cultures +ve in 9/26 (35%) cases - *S. pneumoniae* 8- *S. pyogenes* 1. All *S. pneumoniae* isolates typed were serotype 5. 17 (59%) had received antibiotics before admission to hospital.	CXR ± CT scans performed.	Median peripheral WBC Count = 14.7 × 10^9^/L.	Chest tube drainage 21 (72%), including urokinase in 6 (21%). VATS 1 (3%).	Median febrile days before presentation = 5 days. Median febrile days in hospital = 6 days. Median chest drainage time = 7 days. Median hospital LOS = 14 days. Median (IQR) antibiotic days = 13 (7) days. No deaths.	NP diagnosed by multiple thin-walled cavities within lung consolidation on CXR or lung tissue liquefaction on CT scan. Jewish ethnicity over-represented by 25 (86%) cases.

*BAL* bronchoalveolar lavage, *BPF* bronchopleural fistula, *CAP* community-acquired pneumonia, *CNLD* chronic neonatal lung disease, *CONS* coagulase negative staphylococcus, *CRP* c-reactive protein, *CT* computed tomography, *CXR* chest xray, *ECMO* extracorporeal membrane oxygenation, *Hib Haemophilus influenzae* type b, *IQR* interquartile range, *IU* international units, *LDH* lactate dehydrogenase, *LOS* length of stay, *mmol* millimoles, *MRSA* methicillin-resistant *Staphylococcus aureus*, *MSSA* methicillin-resistant *Staphylococcus* aureus, *No* number, *NP* necrotizing pneumonia, *NR* not reported, *PCV7* 7-valent pneumococcal conjugate vaccine, *PID* primary immunodeficiency (1 had Schwachman-Diamond syndrome, 1 chronic granulomatous disease), *PFT* pulmonary function tests, *PPE* parapneumonic empyema, *PTX* pneumothorax, *PVL* Panton-Valentine leucocidin, *SD* standard deviation, *US* ultrasound, *VATS* video-assisted thoracoscopy, *WBC* white blood cell

**Table 2:** Retrospective studies in children with necrotizing pneumonia secondary to *Streptococcus pneumoniae* infection

Study	Subjects	*S. pneumoniae* serotypes	Imaging results	Management	Course	Comments
Hsieh et al. [39] Taiwan. Single tertiary center. 5 yr. review May 1998–July 2003.	No. = 15. 2 (13%) males. Median age 49 mths (Range 9–85 mths).	10 isolates available for serotyping. Serotype 14 (50%) 3 (30%) 6A (10%) 18C (10%). 11 isolates available for penicillin MICs. 7 (64%) had non-penicillin (MIC ≥1 mg/L) susceptibility.	CXR + CT scans performed. Lungs involved - right 10 (67%) left 5 (33%) multi-lobar 5 (33%). PPE 14 (93%) PTX 1 (7%)	Procedures for PPE 14 (93%). Chest tube drainage only 6 (43%). VATS 8 (57%).	Median febrile days before presentation = 4 days (Range 2–11 days). Median febrile days in hospital = 9 days (Range 4–30 days). Median hospital LOS = 18 days (Range 5–40 days). Mean (SD) antibiotic days for 14 survivors = 24 (8) days (Range 12–38 days). 1 died from HUS and pulmonary gangrene.	Diagnosis of NP made on a median of 6 days (range 1–12) after admission. 13 (87%) received cefotaxime or ceftriaxone 10 (67%) received vancomycin 7 (47%) received both vancomycin and a cephalosporin together as directed therapy.
Fretzayas et al. [40] Greece. Single tertiary center. 6 yr. review January 1999–December 2004.	No. = 10. 5 (50%) males. Mean age 3.1 yrs. Representing 1.3% of all cases of lobar pneumonia in children aged <14 yrs. and 20% of bacteraemic pneumonia cases.	All had +ve blood cultures, 5/7 pleural fluid cultures were also +ve. Serotyping NR.	All had CXR evidence of NP, which was then confirmed by chest CT scans. Epyema present in 7 patients. Chest US aided diagnosis in all 8 cases where it was used.	Empyema drained by thoracentesis or by indwelling continuous intercostal tube drainage. None underwent thoracotomy.	Toxic appearance, persistent fever and abnormal chest findings were seen for a mean 23 days. Hospital LOS 15–35 days. 4 patients examined 1 yr. later had normal spirometry.	NP should be considered in those with continuation of fever, persistently raised or increasing blood inflammatory indices and abnormal auscultatory findings for >5-days, despite antibiotics and especially if bacteremia and empyema are present.
Bender et al. [29] United States. Single tertiary center. 9 yr. review January 1997–March 2006.	No. = 33. 18 (55%) males. Mean age 40 mths. Co-morbidities 2 (6%). Cases increased over time: 5/39 (13%) confirmed PNP cases in 1997–2000 vs 28/85 (33%) in 2001–2006 (OR 3.34, 95%CI 1.11–12).	Blood and pleural fluid isolates. 28/33 (85%) were non-PCV7 vaccine serotypes - 2/5 (40%) 1997–2000- 27/28 (96%) 2001–06. Commonest serotypes - serotype 3 11 (33%) 19 4 (12%) 19A 4 (12%) 1 3 (9%). All penicillin susceptible.	CXR or CT scans performed. PPE 32 (97%).	Procedures for PPE 32 (93%). Chest tube drainage only 19 (59%). Chest tube + surgery 11 (34%).	Nursed in intensive care 18 (55%). Mean hospital LOS = 14 days. 1 (3%) death	May have included a small number of cases with lung abscess. Serotype 3 was most often associated with NP with 11/14 (79%) children with culture-confirmed pneumonia developing cavities. Compared with other serotypes, serotype 3 was more likely to be associated with PNP (OR = 14.7, 95% CI 3.4–86).
Hsieh et al. [32] Taiwan. Single tertiary center. 9 yr. review January 2001–March 2010.	No. = 50. 18 had BPF - 4 (22%) males - mean (SD) age 3.5 (2.7) yrs. co-morbidity 1 (6%) PCV7 available in Taiwan since 2005, but coverage <16% by 2009. None of the PNP cases had received PCV7. Proportions of cases increased between 2004 and 2009.	Blood and pleural fluid isolates (*n* = 50). Commonest serotypes - serotype 14 19 (38%) 19A 10 (20%) 3 7 (14%) 6B 8 (16%). Commonest STs - ST46 7/19 (37%) serotype 14 isolates CC320 (ST320, ST3164) 10/10 (100%) serotype 19A isolates. 6/18 (33%) with BPF had non-penicillin susceptibility and 3/18 (17%) had ceftriaxone. non-susceptibility	CXR or CT scan. Of 18 children with BPF, all had PPE. Bilateral lung involvement 10 (56%).	Procedures for BPF 18 children subset. 9 developed BPF after removal of chest drains. Chest tube + surgery 15 (83%), pulmonary resection in 12. None received fibrinolytics.	18 children with BPF. Median febrile days before presentation = 7 days (Range 4–14 days). Median day of BPF diagnosis = 10 days (Range 1–21 days). Nursed in intensive care 9 (50%). Median febrile days in hospital = 13 days (Range 1–34) days. Median hospital LOS = 32 days (Range 11–62 days). None died.	Study focused upon 18 children with BPF, a more critically ill subset. 6 (33%) had HUS. Multivariate analysis found acute respiratory failure (OR = 8.9, 95%CI 2.6–31) and serotype 19A (OR = 5.0, 95%CI 1.2–22) were independent risk factors for BPF. 11/12 resected lung segments had coagulative necrosis with pulmonary infarction.
Janapatla et al. [25] Taiwan. Single tertiary center. 3 yr. review January 2006–December 2009 Two subsets: a. PNP b. PNP + HUS.	No = 12 PNP. 5 (42%) males. Mean (SD) age = 4.6 (2.4) yrs. (Range 1–8 yrs). PCV7 available in Taiwan since 2005, but coverage <16% by 2009. No. = 17 with PNP + HUS (+ 1 other had empyema). 4 (23%) males. Mean (SD) age = 5.3 (2.8) yrs. (Range 2–10 yrs).	Blood and pleural fluid isolates (*n* = 12). Commonest serotypes - serotype 14 9 (75%) 3 1 (8%) 19A 2 (17%) Commonest STs - ST876 and ST46 in 7/9 (78%) serotype 14 isolates. *S. pneumoniae* isolated from blood (*n* = 16) and pleural fluid (*n* = 2). Commonest serotypes - serotype 14 9 (50%) 3 5 (28%) 19F 2 (11%). Commonest STs - ST46 in 5/9 (56%) serotype 14 isolates ST 180 in 5/5 (100%) serotype 3 isolates.	All cases of NP confirmed by CT scan. Results NR. CXR or CT scan Results NR.	Chest tube + surgery, either VATS or lobectomy, for 10 (83%) children. Chest tube alone 2 (11%) children, including the 1 child with empyema alone Chest tube + surgery, either VATS or lobectomy, for 16 (89%) children	Mean (SD) hospital LOS = 26.2 (9.0) days (Range 16–39 days). Deaths, NR. Mean (SD) hospital LOS = 31.4 (9.0) days (Range 11–65 days). Deaths, NR.	The main focus of this study was on PNP with HUS rather than PNP alone. The 12 children with PNP and 42 with IPD without NP served as controls. 5/18 cases of PNP + HUS had serotype 3 isolates compared with 4/54 IPD + PNP only cases (RR = 3.75, 95%CI 1.1, 12.7). 16/18 HUS isolates carried the *nanC* gene compared with 22/54 control isolates (RR = 2.18, 95%CI 1.52–3.13). As 16/17 children with HUS had PNP, NanC could be a virulence factor for NP too.
Hsieh et al. [34] Taiwan. Six tertiary centers. 2 yr. review March 2010–April 2012.	No. = 57 with PNP. 26 (46%) males. Mean (SD) age: a. Mild necrosis (*n* = 13) 45.4 (16) mths b. Cavitation (*n* = 27) 49.6 (15.7) mths c. BPF (*n* = 17) 39.4 (16.1) mths. No co-morbidities.	Blood and pleural fluid positive testing by culture or PCR. Commonest serotypes in pleural fluid: Serotype - 19A (69%)- 3 (12.5%). 12 (21%) had respiratory viruses detected by PCR.	CXR or CT scan. Mild necrosis (non-enhanced areas on contrast CT scans) (*n* = 13; 23%). Cavitation (including pneumatoceles and abscess) (*n* = 27; 47%). BPF (*n* = 17; 30%).	NR.	Mean (SD) duration of fever and hospital LOS- Mild necrosis: 11.4 (6) and 15.2 (4.5) days; Cavitation: 13.9 (6.5) and 18.3 (6.4) days; BPF: 17.8 (7.6) and 32.8 (16.8) days. No deaths.	Pleural fluid pneumococcal load was significantly higher for serotypes 19A and 3 than other serotypes. Severity of necrosis was associated with pleural fluid pneumococcal load and IL-8 levels.

*BPF* bronchopleural fistula, *CC* clonal complex *CI* confidence interval, *CT* computed tomography, *CXR* chest xray, *HUS* haemolytic uremic syndrome, *IL* interleukin, *IPD* invasive pneumococcal disease, *LOS* length of stay, *MIC* minimum inhibitory concentration, *No* number, *NP* necrotizing pneumonia, *NR* not reported, *OR* odds ratio, *PCR* polymerase chain reaction, *PCV7* 7-valent pneumococcal conjugate vaccine, *PNP* pneumococcal necrotizing pneumonia, *PPE* parapneumonic empyema, *PTX* pneumothorax, *RR* risk ratio, *SD* standard deviation, *ST* sequence type, *US* ultrasound, *VATS* video-assisted thoracoscopy

### Epidemiology

While the incidence of hospitalized childhood pneumonia has declined in countries that have introduced pneumococcal conjugate vaccines (PCVs) [41–43], population rates of complicated pneumonia have increased during the last two decades. Although still relatively uncommon and occurring in <1% of children with CAP [44], the incidence of ‘all-cause’ empyema has increased in the United States (US) [45], and in children aged 2–4 years it rose from 3.7 cases per 100,000 in 1996–1998 to 10.3 cases per 100,000 in 2005–2007 [46], with both pneumococci and *Staphylococcus aureus* the most common pathogens detected. In Utah, the incidence of pediatric pneumococcal empyema increased from 1 per 100,000 in 1993, to 5 per 100,000 in 1999, immediately prior to introducing the 7-valent PCV, before reaching 12.5 per 100,000 in 2007. This increase was due almost exclusively to non-vaccine serotypes [47]. Significant increases in complicated pneumonia have also been reported from the United Kingdom (UK) [48], Europe [49], Australia [44], and Israel [32], although for the latter study only the increase in NP cases was statistically significant, increasing from <0.5 per 100,000 in 2001–2003 to almost 2 per 100,000 in 2009–2010.

Single tertiary pediatric centers have also reported temporal increases in pediatric NP case numbers [18, 22, 28–32]. A French study found that NP contributed to 0.8% of all CAP cases and to 6% of those hospitalized with CAP aged <16 years, with rates of NP complicating hospitalized CAP doubling from 4.5% to 9% between 2006 and 2009 and 2009–2011, respectively [30]. Studies from the UK [18, 22] and the US [28] have reported similar increases in NP cases from the 1990s and into the first decade of this century. Reports of increasing NP rates among children with culture-confirmed (blood, pleural fluid, lung tissue or bronchoalveolar lavage fluid) pneumococcal pneumonia also appeared from centers in Utah [29] and Taiwan [32], where the proportions of children with NP rose from 13% in 1997–2000 to 33% in 2001–2006, and from 45% in 2001 to 81% in 2009, respectively. Reasons for this changing epidemiology are complex, but likely reflect heightened awareness as well as temporal changes in organism and antibiotic prescribing patterns [50].

Cases of NP in children occur globally and have been reported from North America [4, 7, 9, 10, 19, 20, 26, 28, 29], Latin America [26, 38], UK [22, 35], Europe [5, 6, 14, 16, 30, 33, 40], Middle East [3, 12, 27, 31, 37], Asia [8, 11, 17, 21, 24, 25, 32, 36, 39], and Australia [23]. Tables 1 and 2 show that the typical child with either NP or PNP is aged between 2 and 5 years and, except for studies from Taiwan [32, 39], is equally likely to be male or female. Surprisingly, few children have underlying co-morbidities, with asthma the most common chronic disorder identified. While both retrospective [51, 52] and prospective [53–55] studies have identified risk factors associated with complicated pneumonia, only one included cases identified as NP [55]. In this particular study, just 6 of 203 (3%) hospitalized children with CAP had this complication and when analyzing for risk factors they were combined with 52 other children with empyema and another four with lung abscesses. Consequently, in contrast with CAP, it is not possible currently to clearly identify risk factors specifically associated with NP, although some may be biologically plausible. Whenever evaluated, pre-admission ibuprofen was identified as a significant independent risk factor associated with complicated pneumonia, even after adjusting for potential confounding from age, sex, and symptom duration, although confounding by indication could not be excluded [51, 52, 55]. As receiving non-steroidal anti-inflammatory agents might mask both pain and fever, it has been proposed that this might lead to delayed presentation and treatment rather than these agents impairing the child’s immune response to infection, as suggested by other experts [55, 56].

### Microbiology

The most common pathogens associated with NP in children are pneumococci and *S. aureus.* Of the 197 bacterial and fungal pathogens detected in single case reports and case series cited in this review [3, 6–8, 11, 13, 14, 16–24, 26–28, 30, 31, 33, 35–38], 116 (59%) were pneumococci and 45 (23%) were *S. aureus*, including 15 methicillin-resistant *S. aureus* (MRSA) strains. In addition, there were other pediatric cases series restricted to either PNP (*n* = 194 isolates; see Table 2) or *S. aureus* (*n* = 16 isolates) alone [15].

Pneumococci possess multiple virulence factors [57], including its polysaccharide capsule, cell surface proteins, the cell wall, and pneumolysin, a pore-forming toxin [58]. Of these, the most important is the polysaccharide capsule, of which there are at least 98 different serotypes, each capable of shielding the organism from the immune system [59]. Individual serotypes vary in their capacity to colonize, cause local or invasive disease, and express antibiotic resistance genes [57, 60]. Serotypes also vary geographically and change over time, perhaps in response to local ecological competitive pressures from other organisms co-habiting the nasopharyngeal space, as well as selection pressures from antibiotics and PCVs [61]. Indeed, most pneumococcal serotypes associated with PNP (Tables 1 and 2) were not included in the 7-valent PCV, notably serotypes 3, 5, 7F, and 19A, reflecting possible temporal shifts in circulating serotypes and/or vaccine-induced strain replacement disease. Of these, serotypes 3 and 19A were most closely associated with PNP. Serotype 3 has a very thick capsule, which strongly resists opsonophagocytosis and induces a marked inflammatory response, including an intense neutrophilic infiltration with suppurative necrosis [1, 62]. In contrast, serotype 19A strains have greater invasive potential, may have a growth advantage over other pneumococcal serotypes in normally sterile sites, and are often resistant to multiple antibiotics [34, 63].

*S. aureus* also has many virulence factors that help it to instigate colonization, evade host-immune responses, cause tissue injury, and disseminate to other organs. In establishing infection, *S. aureus* expresses surface proteins that mediate adherence and impair local defences, while later in the infection secreted exotoxins disrupt epithelial barriers and immune cell function responses, thereby facilitating tissue invasion [64]. Although it has long been recognized as an important cause of NP, interest in this pathogen was renewed by recent studies linking strains expressing the virulence factor, Panton-Valentine leukocidin (PVL), with severe forms of this disease in previously healthy children and adults [8, 15, 23, 30, 65]. In many cases these PVL-producing isolates were also MRSA strains. PVL is a pore-forming exotoxin, which activates and then destroys immune cells, such as neutrophils, potentially releasing damaging proteases into the surrounding tissues [66]. Of concern, a multi-center French study [65] involving 50 cases of NP caused by PVL-producing strains of *S. aureus* in children and adults aged 1 month to 78 years reported a 56% case fatality rate. Factors associated with mortality were hemoptysis, erythematous rash within 24 h of admission, and peripheral blood leukopenia <3.0 × 10^6^/L. However, this was a non-comparative study and it is therefore difficult to infer whether PVL contributed to pathogenicity. Indeed, whether PVL itself is responsible for the pathological changes seen in NP is controversial. In part, this is because PVL has a strong cell and species specificity, behaving differently in various cell culture and experimental models. For example, neutrophils from humans and rabbits are very sensitive to the effects of PVL in vitro, while those from monkeys and mice are highly resistant [67]. Moreover, while a systematic review and meta-analysis found a strong association between PVL-producing strains of *S. aureus* and skin and soft tissue infections, no such association was seen for invasive infections, including pneumonia [68]. However, this review was limited by including only one small study in children from China, which compared cases of MRSA CAP where there were no significant differences in proportions of PVL-positive (3/22) and -negative (3/33) strains progressing to NP, respectively [21]. Similarly, linking NP with MRSA is also contentious. Many of the observational studies reporting an association between invasive disease and MRSA are from the US, where the PVL-producing USA300 MRSA clone predominates, while in Europe, Australasia, and elsewhere, there are many different MRSA strains in circulation [69]. Furthermore, a recent case-control study of 133 French children and adults with PVL-positive strains of *S. aureus* NP found no evidence for increased clinical severity in those with MRSA infections [70]. Consequently, there are substantial gaps in our knowledge concerning the pathogenesis of *S. aureus* NP and it is likely other cytotoxins play an important role. Indeed, attention has been focused recently on other pore-forming toxins including alpha-hemolysin (or α-toxin), with its proposed mechanisms of action including activating the NLRP3 inflammasome, resulting in severe alveolar necrosis, and inducing platelet-neutrophil aggregation, which leads to further tissue destruction [71].

In addition to pneumococci and *S. aureus*, other respiratory bacterial and fungal pathogens reported occasionally in studies included in the present review are *Streptococcus pyogenes* (*n* = 5); members of the *S. anginosus* group (*n* = 7); *Haemophilus influenzae* (*n* = 2); *Pseudomonas aeruginosa* (*n* = 3); *Stenotrophomonas maltophilia* (*n* = 1); the anaerobic organism *Fusobacterium nucleatum* (*n* = 2); *Mycoplasma pneumoniae* (*n* = 12); *Legionella pneumophila* (*n* = 1); and *Aspergillus* species (*n* = 1). Unlike in adults, *Klebsiella pneumoniae* is not a common cause of NP in children, and in contrast with simple pulmonary abscesses, oral anaerobes are reported rarely [1]. However, it should also be acknowledged many case series may not have undertaken anaerobic cultures. Nevertheless, the adult experience is that anaerobes play a minor role [2].

Viruses, even those associated with outbreaks of severe adenovirus pneumonia, are rarely the sole cause of NP [72]. In contrast, the association of respiratory syncytial virus (RSV) and influenza virus infections with increased nasopharyngeal colonization by pneumococci and *S. aureus* and greater risk of secondary bacterial CAP with enhanced severity is well recognized [73–77]. However, few studies of NP in children have collected information on respiratory viruses. This is surprising given the consistent reports of influenza-like illnesses occurring immediately prior to NP caused by PVL-producing strains of *S. aureus* [53, 65]. There are single case reports of NP associated with either RSV or influenza A alone [10, 12], or in association with MRSA [6], or pneumococci [7], while studies systematically testing for respiratory viruses are limited. In one series of 57 children with PNP, 12 (21%) had respiratory viruses (rhinovirus, *n* = 5; influenza, *n* = 4; parainfluenza, *n* = 2; human coronavirus NL63, 1) detected in their upper airways by polymerase chain reaction (PCR) assays [34]. Another small study [25] reported that 6 of 18 children with PNP complicated by the hemolytic uremic syndrome (HUS) had positive viral cultures (influenza, *n* = 3; and one each of adenovirus plus enterovirus, parainfluenza, and human metapneumovirus). The mechanisms associated with viral–bacterial interactions within the respiratory tract are complex, multi-layered, and incompletely understood. While beyond the scope of this review, viruses can decrease bacterial clearance by disrupting the respiratory epithelial barrier, impairing mucociliary function, increasing bacterial adherence by upregulating adherence proteins, and modulating immune function by interfering with critical components of the innate immune system, downregulating macrophage phagocytosis, decreasing neutrophil intracellular killing, and inducing apoptosis [78, 79]. How such interactions predispose to CAP, including NP, are still unknown, but important to understand when devising public health prevention strategies and seeking to optimize individual patient management.

### Pathology

Histopathological findings in autopsy and surgical lung specimens from adults and children with NP are characterized by necrosis of lung parenchyma, which was thought primarily to be a vascular process triggered by infection leading to vasculitis, activation of the coagulation system and thrombotic occlusion of intrapulmonary vessels accompanied by cavity formation [2, 80]. However, in all age groups intense suppuration is also seen; it is postulated that direct cytotoxic effects from bacterial toxins and secondarily induced intense cytokine-mediated inflammatory responses,(including interleukin-8 mediated neutrophil recruitment, activation and release of proteolytic enzymes) also contribute to tissue injury and destruction [1, 32, 34, 37]. The mixture of coagulation and liquefactive lung necrosis leads to one or more thin-walled cavities that can form pneumatoceles from the one-way passage of gas [5], or evolve into pulmonary abscesses [81]. PPE and empyema are also common and if necrotic regions extend to the pleura, BPF may form, resulting in persistent gas leaks from communication between the lung and pleural space, especially following surgical interventions [32]. Rarely, the ischemia secondary to simultaneous thromboses of multiple intrapulmonary vessels can result in pulmonary gangrene of an entire lobe late in the disease course—this is a phenomenon that is recognized more commonly in adults [2, 39].

### Clinical features

Most children with NP are young, aged <5 years, and have been healthy previously (Tables 1 and 2). The clinical features are those of pneumonia with fever, cough, chest pain, tachypnea, and localizing chest signs that may include percussion dullness, decreased breath sounds, and/or bronchial breathing. Symptoms may have been present for several days before presentation (Table 1) and despite receiving appropriate therapy the children are often disproportionately sick with persistent fever, respiratory distress and clinical and/or radiographic signs of a non-responding or progressive pneumonia [40, 48, 79]. Most (63–100%) have an accompanying PPE or empyema, while BPF diagnosed by either a pneumothorax on chest radiograph or persistent (>24 h) gas leaks from chest tubes [22, 32] are also a frequent (17–67%) complication (Tables 1 and 2). A much rarer complication is HUS that leads to a microangipathic hemolytic anemia, thrombocytopenia, and acute renal failure within a few days of symptom onset. Taiwanese studies [25] report PNP to be associated with HUS, especially from serotype 3 and other pneumococcal strains possessing the neuraminidase gene, *nanC*, whose enzyme product cleaves sialic acid residues on the surfaces of red blood cells, platelets and endothelial cells.

NP is frequently associated with elevated inflammatory markers, including high peripheral white blood cell counts in PNP and C-reactive protein levels exceeding 100 mg/L, while mild-to-moderate anemia and hypoalbuminemia are also common (Table 1). Pleural fluid typically has the features of an empyema with either frank pus, visible organisms on Gram stain, or increased white blood cells (≥15.0 × 10^6^/L, predominantly neutrophils), and is characterized by pH <7.20, protein >30 g/L, glucose levels <2.2 mmol/L, and lactate dehydrogenase concentrations often ≥1000 U/L, the latter reflecting lung parenchymal injury [82]. In contrast, pleural fluid in patients with *M. pneumonia*-associated NP have high protein content, normal glucose concentration and often a predominance of lymphocytes [17].

### Diagnosis

The diagnosis of NP should always be considered in a child with pneumonia who remains unwell, despite at least 72 h of appropriate antibiotics. This is particularly important if there is evidence of BPF or if a PPE or loculated empyema is present, which has undergone drainage or has been surgically managed without improvement [35, 40, 80]. Extra-pulmonary sites of infection, such as in the skin and soft tissues or musculoskeletal systems, should be sought. Occasionally children with NP can deteriorate rapidly following their initial presentation with features of severe sepsis, including septic shock, multi-organ failure, and hypoxic respiratory failure [23]. The onset of pulmonary hemorrhage, hemoptysis, an erythematous rash, and declining peripheral white blood cell counts are all ominous signs, and are associated most strongly, but not exclusively, with *S. aureus* infections [64]. When NP is being considered there are several radiographic and microbiologic studies that can help make the diagnosis and identify the causative organism respectively.

### Radiographic diagnosis

**Fig. 1:**
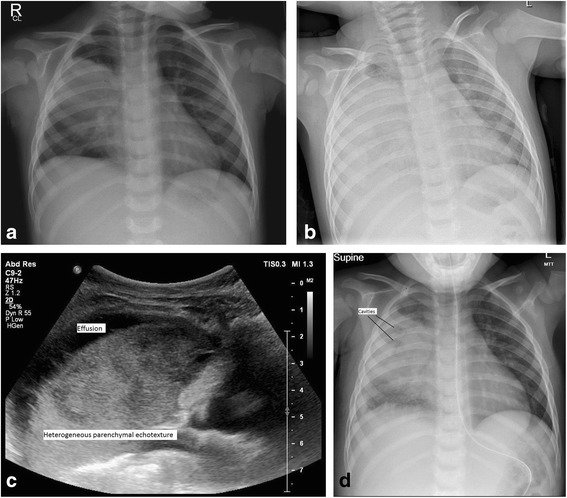
A 2-year-old child presenting with 4 days of fever, cough, and intermittent grunting respirations. Pleural fluid tested positive for pneumococcal antigen. **a** Initial plain chest radiograph showing right mid-zone airspace opacity, consistent with pneumonia. **b** Plain chest radiograph 24 h later revealing a large right-sided pleural effusion. **c** Lung ultrasonographic image taken immediately afterwards depicting the pleural effusion and heterogeneous parenchymal echo texture, consistent with an underlying necrotizing pneumonia. **d** Plain chest radiograph 4 days later following thoracoscopy and removal of chest drains demonstrates right upper zone cavities within the region identified previously by ultrasonography

**Fig. 2:**
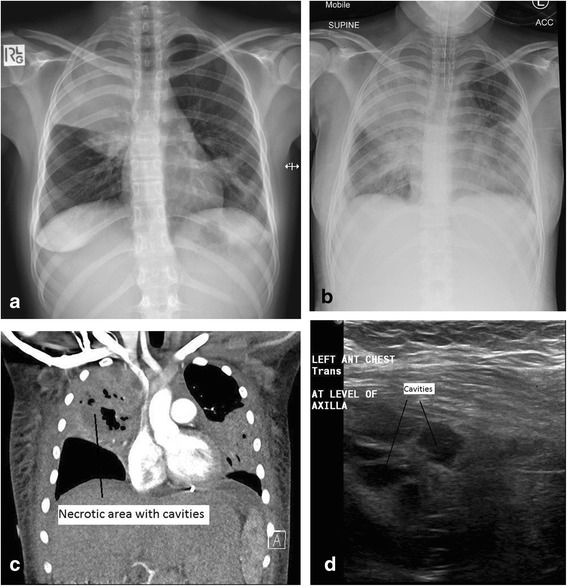
A 14-year-old child presenting with 5 days of fever, cough, and sore throat. Over the next 24 h the patient developed progressive respiratory and multi-organ failure. *S. constellatus* (of the *S. anginosus* group) was cultured from pleural fluid collected on the second day of hospitalization. **a** Initial plain chest radiograph showing a dense right upper zone airspace opacity and lingula airspace changes, consistent with multi-focal pneumonia. The following images were performed 24 h later. **b** Plain chest radiograph with the child intubated and ventilated revealing cavitation in the right mid to upper zones, pleural effusion and more general airspace changes bilaterally. **c** Computed tomography (CT) scan, coronal view, demonstrating non-enhancing area (necrotic) thin-walled cavities within the right upper lobe and lingula. **d** Lung ultrasonographic image displaying thin-walled cavities in the lingula region of the left lung

The diagnosis of NP is generally made by chest imaging studies (Figs. 1 and 2).

#### A. Chest radiographs

Although chest radiographs will demonstrate the underlying pneumonia, accompanying PPE, and if there is a resulting mediastinal shift, this investigation is only diagnostic in 27–41% of children with NP where one or more small lucencies or pneumatoceles (Fig. 1a,b and d; and Fig. 2a, b) are seen at a median of 4–8 days following hospitalization [30, 83]. The poor sensitivity of chest radiographs results from the cavitory lesions being filled with fluid following liquefaction necrosis that has the same density as the adjacent consolidated lung. The lesions become more visible later in the course of NP when the necrotic fluid drains into communicating bronchi and is replaced by gas [83].

#### B. Computed tomography (CT)

Contrast-enhanced chest CT scans are more sensitive than chest radiographs and have become the standard imaging procedure for making the diagnosis of NP, evaluating the lung parenchyma changes not visible on plain chest radiographs, and determining whether any underlying congenital lung malformations exist [16, 80, 81]. The key diagnostic features are poor or absent vascularity, loss of pulmonary architecture, and finally cavity formation. Multiple small, gas- or fluid-filled, thin-walled cavities are usually seen initially, often involving just a single lobe. These are non-enhancing, indicating reduced perfusion (Fig. 2c), while loculated fluid or gas within the pleural cavity is also generally present. As the lung undergoes further liquefactive necrosis, the multiple small cavities may coalesce and form larger cavities, including gas-filled pneumatoceles that develop in the later stages of NP. Nevertheless, controversies over interpretation of radiographic findings remain. These include what defines a cavity in terms of its wall thickness, where, for example, lung abscesses evolving from NP have thick walls in addition to contrast enhancement and often a gas-fluid level [14, 84, 85], while differentiating between pleural and parenchymal spaces can be difficult when a PPE is adjacent to areas of liquefied lung. CT scans are also not without other limitations, including exposure to ionizing radiation, cost, feasibility in critically ill patients, and availability in poorly resourced settings.

#### C. Sonography

Ultrasonography is the primary imaging modality to evaluate the pleural space in children with complicated pneumonia [86]. It also identifies consolidated lung [87] and when combined with color Doppler to detect hypoperfused regions of the lung a retrospective study [88] found good correlation (*r* = 0.704) with CT scan results in 236 children with CAP, 80 of whom had evidence of NP. Lung ultrasonography has attractive features of portability, no radiation exposure, low cost, wide availability and no need for sedation in the young patient. It should therefore be considered when performing a CT scan is neither safe nor feasible, and where an early diagnosis of NP will influence patient management. Detecting hypoechoic lesions (Figs. 1c and 2d) or impaired regional perfusion using combined lung ultrasonography and color Doppler predicts the high likelihood of underlying NP and pneumatocele formation [88]. Nevertheless, this imaging technique is still operator-dependent and collateral circulation in areas of necrosis and ventilation-perfusion mismatch within consolidated lung may also affect sensitivity and specificity respectively.

### Microbiologic diagnosis

Peripheral blood cultures, serological tests for pathogens, such as *M. pneumoniae*, and pleural fluid investigations, including Gram stain, culture and culture-independent tests (such as PCR or rapid antigen assays) are useful in establishing an etiologic cause. Of these, pleural fluid provides the greatest microbiologic yield (Tables 1 and 2), especially when using sensitive molecular diagnostic or rapid antigen techniques. Pleural fluid can be obtained at the time of inserting an intercostal tube to drain the pleural space, at the time of surgery, or even by intercostal needle aspiration if a PPE is present. Sputum cultures are generally not achievable and are unreliable as potential pathogens, such as pneumococci, are found frequently in healthy children. Similarly, while identifying viruses in respiratory secretions is important in gaining further understanding of the pathogenesis of NP, and possibly for infection control measures, positive results are unlikely to have a major influence on management (such as the discontinuation of antibiotics), as detecting viruses does not mean they caused the underlying NP [89].

The studies of children with NP displayed in Table 1 show positive microbiology results in 8–55% of cases and demonstrate the challenges of making a microbiologic diagnosis. Many children (44–100%) had received antibiotics before hospitalization, which in one study [31] reduced positive culture results from 64% in those without antibiotics to 22% in those who had received them before hospitalization. Moreover, clinicians relied heavily upon traditional culture methods to identify the likely causative pathogen. The studies with the greatest microbiologic yields came from the US (48%), France (51%) and the UK (55%) [28, 30, 35]. However, the US study [28] included four cases with positive blood cultures for coagulase-negative staphylococci that are likely contaminants and of the 13 cases with positive pneumococcal cultures, an unknown number originated from sputum. Importantly, this study found that 5 of 50 pleural fluid samples produced positive pneumococcal antigen results, highlighting the usefulness of this test. In the French study [30], of the 13 children with *S. aureus* NP, 7 had positive blood cultures, 5 had positive pleural fluid cultures and 1 each had positive protected-brush specimen or bronchoalveolar lavage cultures for this organism, respectively. Of the 7 children with PNP, 2 had positive blood cultures, while all 7 had pneumococci cultured from their pleural fluid. In the same study, the importance of testing pleural fluid was shown when *F. nucleatum* was detected by 16S PCR testing [30]. The small, select UK surgical study also had a relatively high diagnostic yield from PCR and culture of pleural fluid, despite all having received antibiotics for several days previously [35].

Thus some of the diagnostic difficulties associated with prior antibiotics and low organism loads in sterile sites may be addressed by sensitive molecular techniques within the laboratory [90, 91]. PCR is being employed for detecting pneumococci in pleural fluid [34, 35], and while not yet an established test for blood specimens, this offers hope for the future [92]. Similarly, rapid diagnostic tests against a broad range of respiratory pathogens are undergoing development, although differentiating carriage from disease when sampling from non-sterile sites remains a major limitation with non-invasive tests [93].

### Differential diagnosis

A broad range of disorders cause pulmonary cavities (Table 3). In children with NP, important differential diagnoses include other infections associated with lung cavities [84], secondarily infected congenital lung abnormalities and traumatic pseudocysts [94]. Underlying, unsuspected primary immunodeficiency is uncommon in patients with NP [28], but should be suspected in those with a history of recurrent severe or persistent infections, especially if caused by unusual or opportunistic pathogens or by catalase positive organisms when chronic granulomatous disease is being considered [Table 3]. Differentiating NP from a lung abscess is especially important, as the underlying causes and treatment can differ [15, 95]. A simple lung abscess often runs a more indolent course with fever and cough that may have been present for weeks, but this becomes complicated if investigations are delayed, as multiple thin-walled cavities in NP may coalesce to form one or more contrast-enhancing, thick-walled abscesses containing gas-fluid levels within areas of consolidated lung.

**Table 3:** Causes of pulmonary cavities in children

Primary infectious causes
Bacterial infections
- Necrotizing pneumonia
- Lung abscess
- Septic pulmonary emboli
Uncommon bacterial infections
*- Actinomyces* spp
*- Nocardia* spp
*- Burkholderia pseudomallei* (melioidosis)
Mycobacterial infections
- *Mycobacterium tuberculosis*
- Non-tuberculous mycobacteria spp
Fungal infections
- *Aspergillus*
- Mucormycoses
- Other – eg. *Cryptococcus* spp., *Pneumocystis jirovecii*
Helminthic infections
- *Echinococcus* spp
- Other – eg. *Paragonimus westermani*
Secondarily infected congenital lung malformations
- eg. bronchogenic cysts, congenital pulmonary adenomatoid malformation and bronchopulmonary sequestration
- or a congenital diaphragmatic hernia with an intercurrent respiratory illness
Other causes
Traumatic pseudocysts
Underlying immunodeficiency^a^
- eg. chronic granulomatous disease or hyper IgE syndrome with recurrent infections and pneumatoceles persisting >1 yr
Malignancy
- eg. primary germ cell tumor, lymphoproliferative disorders Langerhan cell histiocystosis
Vasculitis syndromes
- eg. Wegener granulomatosis, Churg-Strauss syndrome

^a^Uncommonly, the initial presentation of chronic granulomatous disease may be with necrotizing pneumonia in a previously well child. This should be considered when the isolated pathogen is one of the catalase +ve organisms associated with pulmonary disease in this disorder (eg. *S. aureus, Aspergillus, Pseudomonas, Burkholderia or Nocardia* species)

### Management

Management of NP relies upon expert opinion, and results of retrospective observational studies from mainly single centers, as to date no randomized-controlled trials comparing different treatments have been performed. A multi-disciplinary team of pediatric respiratory physicians, intensivists, thoracic surgeons, and infectious diseases experts is often required. The overarching aims are to control and ultimately reverse the pathobiologic changes associated with NP. These include providing supplemental oxygen to relieve hypoxia, ensuring adequate analgesia to reduce pleuritic pain and improve ventilation, administering prolonged antibiotic therapy, and decreasing any mass effect or intrathoracic pressure by draining gas and/or intrapleural fluid [50, 80, 81, 86, 96]. Correcting fluid and electrolyte abnormalities and attention to nutrition, including managing hypoalbuminemia, is also important. Some children will require circulatory and ventilation support, while occasionally extracorporeal membrane oxygenation (ECMO) is used in those with refractory hypoxemic respiratory failure [19, 23, 28]. Severely ill children with suspected or proven *S. aureus* or *S. pyogenes* infection—especially with bilateral lung involvement, pulmonary hemorrhage, or impaired circulation—may also benefit from high-dose intravenous (IV) immunoglobulin infusion (2 g/kg), which is repeated after 48 h if there is no improvement [66, 97].

### Antibiotics

A prolonged course of IV antibiotics is the cornerstone of therapy. The initial choice of antibiotics in otherwise healthy, fully immunized children should be the same as for empyema and cover gram positive organisms, especially pneumococci, *S. aureus* and *S. pyogenes* [86], taking into account local epidemiologic and microbiologic data. Consequently, the recommended first-line treatment of IV penicillin or ampicillin for children hospitalized with severe but uncomplicated CAP [98, 99] will need broadening to include beta-lactam anti-staphylococcal antibiotics, such as oxacillin or flucloxacillin [28, 33]. Treatment can then be tailored according to microbiological results, although these may only be positive in 8–55% of cases (Table 1). When suspicion of MRSA is high (eg. local prevalence >10%, ethnicity, recent personal or household history of skin infections) or if it is confirmed by culture, antibiotics should be directed against this specific pathogen. Importantly, vancomycin penetrates poorly into lung parenchyma [100] and treatment failures can occur in up to 20% of MRSA pneumonia when used as monotherapy [101]. Thus, until MRSA is confirmed, a beta-lactam anti-staphylococcal antibiotic should be part of the treatment regimen. While high-level evidence is lacking, the addition of agents such as linezolid, clindamycin, or rifampicin capable of inhibiting protein synthesis (including toxin production) may result in better outcomes for those with *S. aureus* or *S. pyogenes* infections [21, 99]. When NP complicating an *M. pneumoniae* infection is suspected, a macrolide such as IV clarithromycin or azithromycin is added [24]. However, these agents should not invariably replace antibiotics active against pneumococci and *S. aureus*, given the high rates of mixed infection associated with *M. pneumoniae* pneumonia, frequent negative microbiology results in patients with NP, and increasing levels of macrolide resistance in respiratory bacterial pathogens [102]. Finally, the initial empiric antibiotic therapy may need to provide extended gram negative coverage by including a third or fourth generation cephalosporin if the child is unimmunized against *H. influenzae* type b (Hib), immunocompromized, or if the infection was hospital-acquired.

The optimal duration for antibiotic treatment of NP is unknown. The median length of antibiotic courses in case series listed in Table 1 range from 13 to 42 days, with 3 of the 5 studies providing these data reporting a median antibiotic course duration of 28 days [28, 33, 36]. Switching from IV to oral antibiotics is appropriate once the child is afebrile for at least 24 h and no longer showing signs of sepsis, their respiratory distress is resolving, enteral feeds are being tolerated, and inflammatory markers are declining [103]. At this point antibiotics are continued for at least another 10–14 days, a recommendation that aligns with consensus guidelines for PPE and empyema complicating pediatric CAP [86, 99].

### Surgical management

While it is recommended that surgical intervention is kept to a minimum to avoid risking BPF [14, 28, 30, 33, 36, 38, 40], this is not always possible if a large pyopneumothorax, tension pneumatocele and/or loculated empyema are already present, especially if they persist or lead to mass effects causing hemodynamic instability and further compromise of ventilation. Moreover, necrotic lung may only be discovered at the time of surgery [8179]. Experienced pediatric surgeons are crucial for management, especially as surgical intervention has become less common in children with NP from Europe, the UK, and US (Table 1), and is particularly challenging because of risk of bleeding when ECMO is being used.

Surgery has two principal aims. The first is to manage concomitant pleural disease involving moderate to large PPE, multi-loculated empyema and persistent gas leak from BPF. This also provides an opportunity to collect pleural fluid for microbiologic testing. Chest tube drainage alone for large PPEs and pyopneumothorax may suffice, but for those with loculated empyema, chest tube insertion and installation of intrapleural fibrinolytics is an option preferred by some experts [50]. However, chest tube drainage >7 days and fibrinolytics may also be associated with increased risk of BPF developing [14, 28, 33, 35, 96] and can fail in 10–30% of empyema cases [104, 105]. Surgical intervention with either video-assisted thoracoscopic surgery or mini-thoracotomy to debride pyogenic material around the lung (decortication), breakdown loculations, and remove pus may be required when fever, signs of sepsis and/or respiratory distress continue, despite chest tube insertion with or without fibrinolytic therapy and repeat imaging shows persistent or increasing intrapleural collections [86]. However, if pleural disease is minimal, the aforementioned symptoms may be from the underlying NP and continuing IV antibiotics without surgery is recommended. BPF with pyopneumothorax may occur spontaneously in NP or be related to surgical intervention, including protracted chest tube placement, and cause significant morbidity [28, 33, 35]. If gas leak continues despite prolonged drainage, surgery may be required to seal the fistula with fibrin glue or insertion of a muscle flap [35].

The second aim of surgery is managing progressive lung parenchymal necrosis. This involves segmental or lobar resection or pneumonectomy and is rarely required in children. Indications for surgery include massive hemoptysis, large or multiple tension pneumatoceles, and pulmonary gangrene where cavities or subsequent abscesses exceed 50% of the involved lobe [2, 5, 106].

### Course and outcome

Children with NP usually have a prolonged hospital stay. The median length of stay in cases listed in Table 1 ranged from 12 to 30 days. Not surprisingly, those undergoing surgery had a longer stay in hospital than those managed conservatively [36, 106].

Despite receiving appropriate antibiotic therapy and drainage of any accompanying PPE and empyema, children with NP often have intermittent fevers for several days. This can be part of the natural history of the infection, where poor penetration of antibiotics into hypoperfused regions of the lung and into cavitating lesions leads to delayed bacterial clearance, tissue necrosis, and ongoing inflammation [2]. Nevertheless, when the child remains toxic with persistent fevers, ongoing respiratory distress and supplemental oxygen requirement, accompanied by sustained elevations of inflammatory markers, further evaluations are required to determine whether primary source control within the thoracic cavity has been achieved, if other foci of infection exist (eg. osteomyelitis/septic arthritis, infective endocarditis/pericarditis, deep-seated abscesses or intravascular line infections) or venous thromboses have developed. In cases without a microbiologic diagnosis, incorrect antibiotic choices and the possibility of resistant organisms must also be considered.

Additional complications worth highlighting include HUS that may emerge during the course of PNP, particularly in Taiwanese children [25, 32], and the early or late-onset of life-threatening tension pneumatoceles and tension pneumothorax [4, 26]. Tension pneumatoceles arise from ball-valve mechanisms, trapping gas within the cavity, while a late tension pneumothorax may occur from an unrecognized small BPF leading to a low-grade leak and gradual accumulation of gas within the pleural cavity under pressure [26]. Both complications need urgent surgical intervention and thus experts advise that children with residual cavities should have repeat chest radiograph 2 weeks following discharge, or sooner if they develop signs of respiratory distress.

Unlike adults where case fatality rates for NP can range from approximately 40–50% [15, 65, 70, 101], deaths in children are uncommon despite them often being critically ill and requiring management in intensive care units. Although deaths are reported in single case reports and small case series, these articles are overrepresented by *S. aureus* infections and may be subject to publication bias [4, 15, 23, 72]. Of the 8 studies and 283 patients displayed in Table 1, only two deaths were reported, both from a single center (2/36; 5.5%) where 24 (67%) underwent thoracotomy [37]. Similarly, deaths were also uncommon in PNP (Table 2), where there were just two deaths, including 1 from HUS and pulmonary gangrene, amongst 72 patients from case series where fatalities were reported [29, 39]. In the largest case series published to date, most patients made a full clinical recovery within 2 months of hospitalization, although eight (10%) were readmitted within 2 weeks of discharge, mostly because of ongoing fever [28]. In this study, 12 children also had follow-up spirometry, eight had normal results, but mild obstructive defects were observed in 3 children and mild restriction in another, findings similar to those hospitalized with severe CAP [107]. In most cases, chest radiographs and CT scans normalized or improved markedly by 5–6 months [16, 28, 33, 35, 36, 83, 106].

Consequently, in addition to reviewing children and repeating their chest radiograph 2 weeks following discharge, the authors recommend further clinical reviews and chest radiographs at 6–8 weeks and 6 months as a minimum. In those old enough to perform spirometry, this investigation should be done at discharge and again at 6 months. Referral to a pediatric respiratory physician for further investigations is indicated when symptoms and radiographic abnormalities persist beyond the expected time frames of 2 months [28] and 6 months [16, 28, 33, 35, 36, 83, 106], respectively.

### Prevention

Until there is a greater understanding of why NP occasionally complicates pneumonia, its prevention will depend upon reducing CAP and its severity. Efforts should focus upon children aged <5 years who have the highest incidence of CAP [108]. Indeed, since the year 2000, deaths from CAP in this age group have declined >50% globally, falling below one million in 2015 [109]. Decreases in CAP are attributed to a combination of improved housing, water supply and hygiene, better indoor air quality, reduced parental tobacco smoking, increased education, breastfeeding rates and nutrition, and greater healthcare access and vaccine uptake [110]. Vaccines contributing to this success globally are pertussis, measles, Hib, and pneumococcal conjugate vaccines [111].

Despite these advances, NP cases have increased in the last two decades [18, 22, 28–32]. In part, this may be from increased physician awareness and requesting chest CT scans in children with severe, refractory, or complicated pneumonia. Temporal changes in pathogens, including pneumococcal serotypes and *S. aureus*, may also be important. It is therefore encouraging that since replacing the 7-valent PCV in the US and France with a 13-valent formulation containing many of the serotypes associated with NP, rates of PPE/empyema in children fell rapidly by 37–53% [112, 113]. Whether this decline is also true for NP is unknown.

Nevertheless, replacement disease by non-vaccine pneumococcal serotypes remains a potential problem [108], and an effective vaccine against *S. aureus* continues to be elusive [114]. However, efforts continue to improve performance and acceptability of pertussis and influenza vaccine formulations [115, 116], and candidate vaccines against *S. pyogenes* and RSV are undergoing clinical trials [117, 118]. Maternal antenatal immunization with pertussis and influenza provide early protection in very young infants and are gaining wider acceptance [119, 120], while novel RSV vaccines administered during pregnancy and to older infants are also being evaluated [121]. Hopefully, these bundles of social, environmental, and medical interventions will further reduce pediatric CAP and its complications.

## Conclusions

NP is an uncommon but increasingly recognized severe complication of pneumonia in previously healthy young children. The major pathogens are *S. pneumoniae* and *S. aureus* and the diagnosis should be considered when, despite appropriate antibiotics, the child remains febrile and unwell with persistent signs of respiratory distress and pneumonia. Most will have a PPE, empyema and/or BPF that has not improved despite chest drainage or surgical intervention. The diagnosis is confirmed by chest imaging, usually by a CT scan or sonography, while treatment requires prolonged IV antibiotics, which can be changed to oral medication for an additional 10–14 days, once the child is afebrile and clinically stable. Ideally, surgical intervention is kept to a minimum, but this is not always possible if there are mass effects from gas and fluid in the pleural cavity or pulmonary gangrene leading to massive hemoptysis, uncontrolled sepsis, or difficulties with assisted ventilation. Nevertheless, despite its severity, mortality in children is uncommon; the children improve clinically within a couple of months, radiographic changes are largely resolved after 5–6 months, and only a minority are left with mildly impaired lung function. Important targets for future research include identifying host–pathogen interactions leading to disease, improving the microbiologic diagnostic gap, optimizing medical and surgical management, and ultimately preventing this severe complication of pediatric pneumonia.

## Abbreviations

BPF: Bronchopleural fistulae
CAP: Community-acquired pneumonia
CT: Computed tomography
ECMO: Extracorporeal membrane oxygenation
Hib: *Haemophilus influenzae* type b
HUS: Hemolytic uremic syndrome
IV: Intravenous
MRSA: Methicillin-resistant *Staphylococcus aureus*
NP: Necrotizing pneumonia
PCR: Polymerase chain reaction
PCV: Pneumococcal conjugate vaccine
PNP: Pneumococcal necrotizing pneumonia
PPE: Parapneumonic effusion
PVL: Panton-Valentine leucocidin
RSV: Respiratory syncytial virus
UK: United Kingdom
US: United States

## References

[1] Tsai Y-F, Ku Y-H (2012). Necrotizing pneumonia: a rare complication of pneumonia requiring special consideration. Curr Opin Pulm Med.

[2] Chatha N, Fortin D, Bosma KJ (2014). Management of necrotizing pneumonia and pulmonary gangrene: a case series and review of the literature. Can Respir J.

[3] Kerem E, Bar Ziv Y, Rudenski B, Katz S, Kleid D, Branski D (1994). Bacteremic necrotizing pneumococcal pneumonia in children. Am J Respir Crit Care Med.

[4] Al-Saleh S, Grasemann H, Cox P (2008). Necrotizing pneumonia complicated by early and late pneumatoceles. Can Respir J.

[5] Cicak B, Verona E, Mihatov-Stefanovic I (2010). Necrotizing pneumonia in infants. Acta Clin Croat.

[6] Obando I, Valderrabanos ES, Millan JA, Neth OW (2010). Necrotising pneumonia due to influenza a (H1N1) and community-acquired methicillin-resistant *Staphylococcus aureus* clone USA300: successful management of the first documented paediatric case. Arch Dis Child.

[7] Yazer J, Giacomantonio M, MacDonald N, Lopushinsky S (2011). Severe necrotizing pneumonia in a child with pandemic (H1N1) influenza. Can Med J.

[8] Chen J, Luo Y, Zhang S, Liang Z, Wang Y, Zhang T (2014). Community-acquired necrotizing pneumonia caused by methicillin-resistant *Staphylococcus aureus* producing Panton-valentine leucocidin in a Chinese teenager: case report and literature review. Int J Infect Dis.

[9] Pawley B, Smith M, Nickels D (2016). Core curriculum illustration: necrotizing pneumonia and empyema. Emerg Radiol.

[10] Ramgopal S, Ivan Y, Medsinge A, Saladino RA (2017). Pediatric necrotizing pneumonia and review of the literature. Pediatr Emerg Care.

[11] Lu S, Tsai JD, Tsao TF, Liao PF, Sheu JN (2016). Necrotizing pneumonia and acute purulent pericarditis caused by *Streptococcus pneumoniae* serotype 19A in a healthy 4-year old girl after one catch-up dose of 13-valent pneumococcal conjugate vaccine. Paediatr Int Child Health.

[12] Ramoglu MG, Ucar T, Kendirli T, Eyileten Z, Atalay S (2016). Necrotizing pneumonia caused by H1N1 virus in a child with total anomalous pulmonary venous connection after surgery. Acta Cardiol Sin.

[13] McCarthy VP, Patamasucon P, Gaines T, Lucas MA (1999). Necrotizing pneumonia in childhood. Pediatr Pulmonol.

[14] Hoffer FA, Bloom DA, Colin AA, Fishman SJ (1999). Lung abscess versus necrotizing pneumonia: implications for interventional therapy. Pediatr Radiol.

[15] Gillet Y, Issartel B, Vanhems P, Fournet J-C, Lina G, Bes M (2002). Association between *Staphylococcus aureus* strains carrying gene for Panton-valentine leucocidin and highly lethal necrotising pneumonia in young immunocompetent patients. Lancet.

[16] Hodina M, Hanquinet S, Cotting J, Schnyder P, Gudinchet E (2002). Imaging of cavitary necrosis in complicated childhood pneumonia. Eur Radiol.

[17] Wang RS, Wang SY, Hsieh KS (2004). Necrotizing pneumonitis caused by *Mycoplasma pneumoniae* in pediatric patients. Pediatr Infect Dis J.

[18] Ramphul N, Eastham KM, Freeman R, Eltringham G, Kearns AM, Leeming JP (2006). Cavitatory lung disease complicating empyema in children. Pediatr Pulmonol.

[19] Stroud MH, Okhuysen-Cawley R, Jaquiss R, Berlinski A, Fiser RT (2007). Successful use of extracorporeal membrane oxygenation in severe necrotizing pneumonia caused by *Staphylococcus aureus*. Pediatr Crit Care Med.

[20] Kalaskar AS, Heresi GP, Wanger A, Murphy JR, Wootton SH (2009). Severe necrotizing pneumonia in children, Houston, Texas, USA. Emerg Infect Dis.

[21] Geng W, Yang Y, Wu D, Zhang W, Wang C, Shang Y (2010). Community-acquired, methicillin-resistant *Staphylococcus aureus* isolated from children with community-onset pneumonia in China. Pediatr Pulmonol.

[22] McKee AJ, Ives A, Balfour-Lynn IM (2011). Increased incidence of bronchopulmonary fistulas complicating pediatric pneumonia. Pediatr Pulmonol.

[23] Schwartz KL, Nourse C (2012). Panton-valentine leucocidin-associated *Staphylococcus aureus* necrotizing pneumonia in infants: a report of four cases and review of the literature. Eur J Pediatr.

[24] Wang Y, Xu D, Li S, Chen Z (2012). *Mycoplasma pneumoniae*-associated necrotizing pneumonitis in children. Pediatr Int.

[25] Janapatla R-P, Hsu M-H, Hsieh Y-C, Lee H-Y, Lin T-Y, Chiu C-H (2013). Necrotizing pneumonia caused by *nanC*-carrying serotypes is associated with pneumococcal haemolytic uraemic syndrome in children. Clin Microbiol Infect.

[26] Demirel N, Quizon A, Belteton de Leon EL, Reiter J, Colin AA (2014). On the nature of pleural involvement in necrotizing pneumonia: a report of two cases of life threatening late complications. Pediatr Pulmonol.

[27] Cakir E, Gedik AH, Ari E, Ozdemir A, Cakir FB, Uzuner S (2015). Nontuberculous pulmonary cavitary diseases of childhood. Pediatr Infect Dis J.

[28] Sawicki GS, Lu FL, Valim C, Cleveland RH, Colin AA (2008). Necrotising pneumonia is an increasingly detected complication of pneumonia in children. Eur Respir J.

[29] Bender JM, Ampofo K, Korgenski K, Daly J, Pavia AT, Mason EO (2008). Pneumococcal necrotizing pneumonia in Utah: does serotype matter?. Clin Infect Dis.

[30] Lemaitre C, Angoulvant F, Gabor F, Makhoul J, Bonacorsi S, Naudin J (2013). Necrotizing pneumonia in children. Report of 41 cases between 2006 and 2011 in a French tertiary care center. Pediatr Infect Dis J.

[31] Erlichman I, Breuer O, Shoseyov D, Cohen-Cymberknoh M, Koplewitz B, Averbuch D (2017). Complicated acquired pneumonia in childhood: different types, clinical course, and outcome. Pediatr Pulmonol.

[32] Hsieh YC, Wang C-W, Lai S-H, Lai J-Y, Wong K-S, Huang Y-C (2011). Necrotizing pneumococcal pneumonia with bronchopleural fistula among children in Taiwan. Pediatr Infect Dis J.

[33] Krenke K, Sanocki M, Urbankowska E, Kraj G, Krawiec M, Urbankowski T (2015). Necrotizing pneumonia and its complications in children. Adv Exp Med Biol.

[34] Hsieh Y-C, Chi H, Chang K-Y, Lai S-H, Mu J-J, Wong K-S (2015). Increase in fitness of *Streptococcus pneumoniae* is associated with the severity of necrotizing pneumonia. Pediatr Infect Dis J.

[35] Jester I, Nijran A, Singh M, Parikh DH (2012). Surgical management of bronchopleural fistula in pediatric empyema and necrotizing pneumonia: efficacy of the serratus anterior muscle digitation flap. J Pediatr Surg.

[36] Wong KS, Chiu CH, Yeow KM, Huang YC, Liu HP, Lin TY (2000). Necrotising pneumonitis in children. Eur J Pediatr.

[37] Hacimustafaoglu M, Celebi S, Sarimehmet H, Gurpinar A, Ercan I (2004). Necrotizing pneumonia in children. Acta Paediatr.

[38] Macedo M, Meyer KF, Oliveira TCM (2010). Necrotizing pneumonia in children submitted to thoracoscopy due to pleural empyema: incidence, treatment and clinical evolution. J Bras Pneumol.

[39] Hsieh Y-C, Hsiao C-H, Tsao P-N, Wang J-Y, Hsueh P-R, Chiang B-L (2006). Necrotizing pneumococcal pneumonia in children: the role of pulmonary gangrene. Pediatr Pulmonol.

[40] Fretzayas A, Moustaki M, Alexopoulou E, Nychtari G, Nicolaidou P, Priftis KN (2009). Clinical notations on bacteraemic cavitating pneumococcal pneumonia in nonvaccinated immunocompetent children. J Trop Pediatr.

[41] Griffin MR, Zhu Y, Moore MR, Whitney CG, Grijalva CG (2013). U.S. hospitalizations for pneumonia after a decade of pneumococcal vaccination. N Engl J Med.

[42] Jardine A, Menzies RI, McIntyre PB (2010). Reduction in hospitalizations for pneumonia associated with the introduction of a pneumococcal conjugate vaccination schedule without a booster dose in Australia. Pediatr Infect Dis J.

[43] Sgambatti S, Minamisava R, Bierrenbach AL (2016). Early impact of 10-valent pneumococcal conjugate vaccine in childhood pneumonia hospitalizations using primary data from an active population-based surveillance. Vaccine.

[44] Strachan R, Jaffe A (2009). Assessment of the burden of paediatric empyema in Australia. J Paediatr Child Health.

[45] Grijalva CG, Zhu Y, Nuorti JP, Griffin MR (2011). Emergence of parapneumonic empyema in the USA. Thorax.

[46] Grijalva CG, Nuorti JP, Zhu Y, Griffin MR (2010). Increasing incidence of empyema complicating childhood community-acquired pneumonia in the United States. Clin Infect Dis.

[47] Byington CL, Hulten KG, Ampofo K (2010). Molecular epidemiology of pediatric pneumococcal empyema from 2001 to 2007 in Utah. J Clin Microbiol.

[48] Spencer DA, Iqbal SM, Hasan A, Hamilton L (2006). Empyema thoracis is still increasing in UK children. BMJ.

[49] Munoz-Almagro C, Jordan I, Gene A, Latorre C, Garcia-Garcia JJ, Palleres R (2008). Emergence of invasive pneumococcal disease caused by nonvaccine serotypes in the era of 7-valent conjugate vaccine. Clin Infect Dis.

[50] Spencer DA, Thomas MF (2014). Necrotising pneumonia in children. Paediatr Respir Rev.

[51] Byington CL, Spencer LY, Johnson TA (2002). An epidemiological investigation of a sustained high rate of pediatric parapneumonic empyema: risk factors and microbiological associations. Clin Infect Dis.

[52] Francois P, Desrumaux A, Cans C, Pin I, Pavese P, Labarere J (2010). Prevalence and risk factors of suppurative complications in children with pneumonia. Acta Paediatr.

[53] Amorim P, Morcillo AM, Tresoldi AT, Fraga Ade M, Pereira RM, Baract EC (2012). Factors associated with complications of community-acquired pneumonia in preschool children. J Bras Pneumonol.

[54] Elemraid MA, Thomas MF, Blain AD (2015). Risk factors for the development of pleural empyema in children. Pediatr Pulmonol.

[55] Krenke K, Krawiec M, Kraj G, Peradzynska J, Krauze A, Kulus M (2016). Risk factors for local complications in children with community-acquired pneumonia. Clin Respir J.

[56] Voiriot G, Dury S, Parrot A, Mayaud C, Fartoukh M (2011). Nonsteroidal antiinflammatory drugs may affect the presentation and course of community-acquired pneumonia. Chest.

[57] Shenoy AT, Orihuela CJ (2016). Anatomical site-specific contributions of pneumococcal virulence determinants. Pneumonia.

[58] Cillniz C, Amaro R, Torres A (2016). Pneumococcal vaccination. Curr Opin Infect Dis.

[59] Jauneikaite E, Tocheva AS, Jefferies JM (2015). Current methods for capsular typing of *Streptococcus pneumoniae*. J Microbiol Methods.

[60] Woodhead M (2014). Pneumococcal serotypes and respiratory failure: soil or seed?. Eur Respir J.

[61] Linares J, Ardunay C, Pallares R, Fenoll A (2010). Changes in antimicrobial resistance, serotypes and genotypes in *Streptococcus pneumoniae* over a 30-year period. Clin Microbiol Infect.

[62] Grabenstein JD, Musey LK (2014). Differences in serious clinical outcomes of infection caused by specific pneumococcal serotypes among adults. Vaccine.

[63] Reinert RR, Jacobs MR, Kaplan SL (2010). Pneumococcal disease caused by serotype 19A: review of the literature and implications for future vaccine development. Vaccine.

[64] Dayan GH, Mohamed N, Scully IL (2016). *Staphylococcus aureus*: the current state of disease, pathophysiology and strategies for prevention. Expert Rev Vaccines.

[65] Gillet Y, Vanhems P, Lina G (2007). Factors predicting mortality in necrotizing community-acquired pneumonia caused by *Staphylococcus aureus* containing Panton-valentine leucocidin. Clin Infect Dis.

[66] Loffler B, Niemann S, Ehrhardt C (2013). Pathogenesis of *Staphylococucs aureus* necrotizing pneumonia: the role of PVL and an influenza coinfection. Expert Rev Anti-Infect Ther.

[67] Loffler B, Hussain M, Grundmeler M, et al. *Staphylococcus aureus* Panton-Valentine leukocidin is a very potent cytotoxic factor for human neutrophils. PLoS Pathogens 2010;6(1):e1000715. 10.1371/journal.ppat.1000715 PMC2798753 20072612

[68] Shallcross LJ, Fragaszy E, Johnson AM, Hayward AC (2013). The role of the Panton-valentine leucocidin toxin in staphylococcal disease: a systematic review and meta-analysis. Lancet Infect Dis.

[69] Chua K, Laurent F, Coombs G, Grayson ML, Howden BP (2011). Not community-associated methicillin-resistant *Staphylococcus aureus* (CA-MRSA)! A clinician’s guide to community MRSA – its evolving antimicrobial resistance and implications for therapy. Clin Infect Dis.

[70] Sicot N, Khanafer N, Meyssonnier V (2013). Methicillin resistance is not a predictor of severity in community-acquired *Staphylococcus aureus* necrotizing pneumonia – results of a prospective observational study. Clin Microbiol Infect.

[71] Tong SYC, Davis JS, Eichenberger E, Holland TL, Fowler VG (2015). *Staphylococcus aureus* infections: epidemiology, pathophysiology, clinical manifestations, and management. Clin Microbiol Rev.

[72] Vaideeswar P, Bavdekar SB, Jadhav SM, Balan R, Pandit SP (2008). Necrotizing adenoviral pneumonia: manifestation of nosocomial infection in pediatric intensive care unit. Ind J Pediatr.

[73] De Lastours V, Malosh R, Ramaugu K (2016). Co-colonization by *Streptococcus pneumoniae* and *Staphylococcus aureus* in the throat during acute respiratory illnesses. Epidemiol Infect.

[74] Teo SM, Mok D, Pham K (2015). The infant nasopharyngeal microbiome impacts severity of lower respiratory infection and risk of asthma development. Cell Host Microbe.

[75] Cevey-Macherel M, Galetto-Lacour A, Gervaix A (2009). Etiology of community-acquired pneumonia in hospitalized children based on WHO clinical guidelines. Eur J Pediatr.

[76] Elemraid MA, Sails AD, Eltringham GJA (2013). Aetiology of paediatric pneumonia after the introduction of pneumococcal conjugate vaccine. Eur Respir J.

[77] Yu KOA, Randolph AG, Agan AA (2016). *Staphylococcus aureus* α-toxin response distinguishes respiratory virus-methicillin-resistant *S. aureus* coinfection in children. J Infect Dis.

[78] Bosch AATM, Biesbroek G, Trzcinski K, Sanders EAM, Bogaert D (2013). Viral and bacterial interactions in the upper respiratory tract. PLoS Pathog.

[79] Brealey JC, Sly PD, Young PR, Chappell KJ. Viral bacterial co-infection of the respiratory tract during early childhood. FEMS Microbiol Lett 2015;362. 10.1093/femsle/fnv062 25877546

[80] Loizzi M, De Palma A, Pagliarulo V, Loizzi D, Sollitto F (2012). Pulmonary infections of surgical interest in childhood. Thorac Surg Clin.

[81] Thomas MF, Wort A, Spencer DA (2014). Management and complications of pneumonia. Paediatr Child Health.

[82] Ulloa-Gutierrez R (2008). Pneumococcal necrotizing pneumonia and pleural fluid lactate dehydrogenase level. Clin Infect Dis.

[83] Donnelly LF, Klosterman LA (1998). Cavitatory necrosis complicating pneumonia in children: sequential findings on chest radiography. Am J Roentgenol.

[84] Gadkowski LB, Stout JE (2008). Cavitary pulmonary disease. Clin Microbiol Rev.

[85] Agasthian T (2012). Results of surgery for bronchiectasis and pulmonary abscesses. Thorac Surg Clin.

[86] Islam S, Calkins CM, Goldin AB (2012). The diagnosis and management of empyema in children: a comprehensive review from the APSA outcomes and clinical trial committee. J Pediatr Surg.

[87] Shah VP, Tunik MG, Tsung JW (2013). Prospective evaluation of point-of-care ultrasonography for the diagnosis of pneumonia in children and young adults. JAMA Pediatr.

[88] Lai SH, Wong KS, Liao SL. Value of lung ultrasonography in the diagnosis and outcome prediction of pediatric community-acquired pneumonia with necrotizing change. PLoS One 2015;10(6):e0130082. 10.1371/journal.pone.0130082 PMC4472812 26086718

[89] Sly PD, Zar HJ (2017). The spectrum of lower respiratory tract illness in children after pneumococcal conjugate vaccination. Am J Respir Crit Care Med.

[90] De Schutter I, Vergison A, Tuerlinckx D (2014). Pneumococcal aetiology and serotype distribution in paediatric community-acquired pneumonia. PLoS One.

[91] Torrses A, Lee N, Cilloniz C, Vila J, Van der Eerden M (2016). Laboratory diagnosis of pneumonia in the molecular age. Eur Respir J.

[92] Murdoch DR (2016). How to best determine causative pathogens in pneumonia. Pneumonia.

[93] Gadsby NJ, Russell CD, McHugh MP (2016). Comprehensive molecular testing for respiratory pathogens in community-acquired pneumonia. Clin Infect Dis.

[94] Odev K, Guler I, Altinok T (2013). Cystic and cavitary lung lesions in children: radiologic findings with pathologic correlation. J Clin Imaging Sci.

[95] Wheeler JG, Jacobs RF, Cherry JD, Harrison GJ, Kaplan SL, Steinbach WJ, Hotez PJ (2014). Complications of pneumonia. Feigin and Cherry’s textbook of Pediatric infectious diseases.

[96] Pabary R, Balbour-Lynn IM (2013). Complicated pneumonia in children. Breathe.

[97] Kreienbuehl L, Charbonney E, Eggimann P (2011). Community-acquired necrotizing pneumonia due to methicillin-sensitive *Staphylococcus aureus* secreting Panton-valentine leucocidin: a review of case reports. Ann Intensive Care.

[98] Harris M, Clark J, Coote N (2011). British Thoracic Society guidelines for the management of community acquired pneumonia in children: update 2011. Thorax.

[99] Bradley JS, Byington CL, Shah SS (2011). The management of community-acquired pneumonia in infants and children older than 3 months of age: clinical practice guidelines by the Pediatric Infectious Diseases Society and the Infectious Diseases Society of America. Clin Infect Dis.

[100] Le J, Lieberman JM (2006). Management of community-associated methicillin-resistant *Staphylococcus aureus* infections in children. Pharmacotherapy.

[101] Li HT, Zhang TT, Huang J, Zhou YQ, Zhu JX, Wu BQ (2011). Factors associated with the outcome of life threatening necrotizing pneumonia due to community-acquired *Staphylococcus aureus* in adult and adolescent patients. Respiration.

[102] Korppi M, Heiskanen-Kosma T, Kleemola M (2004). Incidence of community-acquired pneumonia in children caused by *Mycoplasma pneumoniae*: serological results of a prospective, population-based study in primary health care. Respirology.

[103] McMullan BJ, Andresen D, Blyth CC (2016). Antibiotic duration and the timing of the switch from intravenous to oral route for bacterial infections in children: systematic review and guidelines. Lancet Infect Dis.

[104] Thomson AH, Hull J, Kumar MR, Wallis C, Balfour-Lynn IM (2002). Randomised trial of intrapleural urokinase in the treatment of childhood empyema. Thorax.

[105] Tuncozgur B, Ustunsoy H, Sivrikoz MC (2001). Intrapleural urokinase in the management of parapneumonic empyema: a randomized controlled trial. Int J Clin Pract.

[106] Lai JY, Yang W, Ming YC. Surgical management of complicated necrotizing pneumonia in children. Pediatric Neonatol 2016 Oct 28. doi: 10.1016/j.pedneo.2016.06.002. [Epub ahead of print]. 10.1016/j.pedneo.2016.06.002 27989426

[107] Grimwood K, Chang AB (2015). Long-term effects of pneumonia in young children. Pneumonia.

[108] Shaughnessy EE, Stalets EL, Shah SS (2016). Community-acquired pneumonia in the post 13-valent pneumococcal conjugate vaccine era. Curr Opin Pediatr.

[109] Lu L, Oza S, Hogan D (2016). Global, regional, and national causes of under 5 mortality in 2000-15: an updated systematic analysis with implications for the sustainable development goals. Lancet.

[110] Izadnegahdar R, Cohen AL, Klugman KP, Qazi SA (2013). Childhood pneumonia in developing countries. Lancet Respir Med.

[111] Williams DJ, Shah SS (2012). Community-acquired pneumonia in the conjugate vaccine era. J Pediatr Infect Dis Soc.

[112] Simonsen L, Taylor RJ, Schuck-Paim C, Lustig R, Haber M, Klugman KP (2014). Effect of 13-valent pneumococcal conjugate vaccine on admissions to hospital 2 years after its introduction in the USA: a time series analysis. Lancet Respir Med.

[113] Angoulvant F, Levy C, Grimprel E (2014). Early impact of 13-valent pneumococcal conjugate vaccine on community-acquired pneumonia in children. Clin Infect Dis.

[114] Dayan GH, Mohamed N, Scully IL (2016). *Staphylococcus aureus*: the current state of disease, pathophysiology and strategies for prevention. Expert Rev Vaccines.

[115] Kilgore PE, Salim AM, zervos MJ, Schmitt HJ (2016). Pertussis: microbiology, disease, treatment, and prevention. Clin Microbiol Rev.

[116] Pica N, Palese P (2013). Toward a universal influenza virus vaccine: prospects and challenges. Annu Rev Med.

[117] Good MF, Batzloff PM (2013). Strategies in the development of vaccines to prevent infections with group a streptococcus. Hum Vaccin Immunother.

[118] Modjarrad K, Giersing B, Kaslow DC, Smith PG, Moorthy VS (2016). The WHO RSV vaccine consultation expert group. WHO report. WHO consultation on respiratory syncytial virus vaccine development report from a World Health Organization meeting held on 23-24 march 2015. Vaccine.

[119] Amirthalingam G, Campbell H, Ribeiro S, Fry NK, Ramsay M, Miller E, Andrews N (2016). Sustained effectiveness of the maternal pertussis immunization program in England 3 years following introduction. Clin Infect Dis.

[120] Munoz FM (2016). Infant protection against influenza through maternal immunization. A call for more immunogenic vaccines. JAMA Pediatr.

[121] Griffiths C, Drews SJ, Marchant DJ (2017). Respiratory syncytial virus: infection, detection, and new options for prevention and treatment. Clin Microbiol Rev.

